# Decrease in Formalin-Inactivated Respiratory Syncytial Virus (FI-RSV) Enhanced Disease with RSV G Glycoprotein Peptide Immunization in BALB/c Mice

**DOI:** 10.1371/journal.pone.0083075

**Published:** 2013-12-23

**Authors:** Gertrud U. Rey, Congrong Miao, Hayat Caidi, Suvang U. Trivedi, Jennifer L. Harcourt, Ralph A. Tripp, Larry J. Anderson, Lia M. Haynes

**Affiliations:** 1 National Center for Immunization and Respiratory Diseases, Division of Viral Diseases, Gastroenteritis and Respiratory Viruses Laboratory Branch, Centers for Disease Control and Prevention (CDC), Atlanta, Georgia, United States of America; 2 College of Veterinary Medicine, Department of Infectious Disease, University of Georgia, Athens, Georgia, United States of America; 3 Emory University Department of Pediatrics and Children’s Healthcare of Atlanta, Emory Children’s Center, Atlanta, Georgia, United States of America; University of Iowa, United States of America

## Abstract

Respiratory syncytial virus (RSV) is a high priority target for vaccine development. One concern in RSV vaccine development is that a non-live virus vaccine would predispose for enhanced disease similar to that seen with the formalin inactivated RSV (FI-RSV) vaccine. Since a mAb specific to RSV G protein can reduce pulmonary inflammation and eosinophilia seen after RSV infection of FI-RSV vaccinated mice, we hypothesized that RSV G peptides that induce antibodies with similar reactivity may limit enhanced disease after subunit or other non-live RSV vaccines. In support of this hypothesis, we show that FI-RSV vaccinated mice administered RSV G peptide vaccines had a significant reduction in enhanced disease after RSV challenge. These data support the importance of RSV G during infection to RSV disease pathogenesis and suggest that use of appropriately designed G peptide vaccines to reduce the risk of enhanced disease with non-live RSV vaccines merits further study.

## Introduction

As the single most important cause of lower respiratory tract infections in the infant and young child, respiratory syncytial virus (RSV) is a high priority target for vaccine development[1,2]. Unfortunately, efforts to develop a safe and effective RSV vaccine have been unsuccessful to date. The first candidate vaccine, formalin-inactivated RSV (FI-RSV), was associated with enhanced disease and also caused two deaths upon subsequent natural RSV infection [3-6]. This occurred in children under two years of age but not older children [3-6], possibly because prior infection patterned for a safe response to later infection. A study in mice found that prior live virus vaccination prevented enhanced disease with the formalin inactivated vaccine [7]. Concern that any other non-live RSV vaccine may also predispose for vaccine enhanced disease upon subsequent natural infection has directed development of RSV vaccines for the RSV naive child away from subunit and inactivated virus vaccines and to live virus vaccines [8]. Subunit and inactivated vaccines have been developed and studied in adults and older children and were not associated with development of enhanced disease, but unfortunately, none has yet been shown to be effective [9-11]. Similarly, multiple attenuated viruses have been developed and evaluated but none has yet been shown to be both safe and efficacious in humans [12-14]. The lack of success in developing RSV vaccines to date and the fact that natural infection provides limited protection from re-infection and disease indicate that the task of developing a safe and efficacious live virus vaccine will be difficult. 

Recent studies of the role of antibodies blocking the activities of the RSV G CX3C chemokine motif suggest a new approach to improving the safety of an RSV vaccine. The RSV G has been shown to modify the immune response to RSV infection in mice, in particular by contributing to vaccine enhanced disease, by inducing pulmonary eosinophils, and increasing production of Th2 cytokines [15-19]. Studies have shown that the RSV G CX3C chemokine motif is an important contributor to RSV G -associated immune modulation and disease pathogenesis [20-23]. Interestingly, an anti-RSV G mAb, mAb 131-2G that blocks RSV G binding to CX3CR1, down-regulated FI-RSV vaccine enhanced inflammation in vaccinated mice when given before RSV challenge [24]. This observation led us to hypothesize that using a vaccine to induce an antibody response that mimics this RSV G mAb might also decrease FI-RSV associated enhanced disease and, more importantly, possibly decrease the risk of enhanced disease after other non-live virus vaccines. In this study, we demonstrate that vaccination with an appropriate RSV G peptide that includes the binding site for mAb 131-2G, and the RSV G CX3C motif, decreases FI-RSV enhanced disease in mice when administered at the same time as FI-RSV vaccination. These data support a role for the RSV G in the challenge virus in the pathogenesis of enhanced disease after FI-RSV immunization and suggest that an appropriately constructed RSV G peptide vaccine might be used with a non-live RSV vaccine to help assure its safety.

## Materials and Methods

### Ethics Statement

The study was performed in accordance with the Guide for the Care and Use of Laboratory Animals of the National Institutes of Health. The protocol was approved by the Centers for Disease Control and Prevention (CDC) Institutional Animal Care and Use Committee (Protocol Number: 1771HAYMOUC). No surgeries were performed. All efforts were made to minimize animal suffering during all procedures performed. 

### Viruses and vaccines

RSV A2 or B1 was propagated in Vero cells (ATCC CCL 881) as previously described [25]. At ~ 80% CPE, the culture medium was removed from the infected cell monolayers, serum-free Dulbecco’s Modified Essential Media (SF-DMEM) (Invitrogen) was added to remove any residual fetal bovine serum and the virus lysate harvested after two freeze-thaw cycles. Formalin-inactivated RSV (FI-RSV) was prepared by incubating 1 part formalin (Sigma Aldrich) with 4,000 parts clarified RSV at 37°C for 3 days, centrifuging the treated virus for 1 h at 50,000 x *g*, 4°C and resuspending the pellet at 1/25 of the original volume in SF-DMEM. The virus was centrifuged and the supernatant precipitated with aluminum hydroxide (4mg/ml) overnight at 4°C. The precipitate was pelleted at 1500 x *g*, 4°C for 15 minutes and resuspended in a 1:100 dilution of the original volume in SF-DMEM. FI-Vero was prepared as described above using uninfected Vero cell lysate.

### Peptides

The following G protein peptides were synthesized and used for immunization: two G protein peptides from the Group A conserved region (amino acids (aa) 163-190) based on an RSV A2 strain aa sequence (GENBANK, accession number P0342)(Table 1) and an RSV CH17 A strain (accession number AAC36325) (Figure S1) aa sequence that includes one amino acid difference, A to D at position 184, from the RSV A2 sequence and one peptide from the Group B conserved region (aa 155-206) based on the RSV B18537 strain aa sequence (accession number P20896) (Figure S1). Sequences from the group A and B conserved sequences encompass aa 164-176 which is conserved among all strains and the CX3C motif at aa 182-186. An I-E^d^-restricted 12-mer peptide from the L protein (aa 393-405) (accession number AAA84898) of RSV was used as a negative control immunogen (Table 1). The peptides were synthesized as previously described [20]. For vaccination studies, all the peptides were conjugated to keyhole limpet hemocyanin (CDC Biotechnology Core Facility, Atlanta).

**Table 1:** The RSV G-A2 peptide sequence and location of the CX3C motif in the G protein.

**Peptide**		**Partial G aa sequence ^*a*^**							
		**155**	**163**					**190**	**206**
G-A2			FHFEVFNFVPCSICSNNPT**CWAIC**KRIP						

^a^ G-A2 peptide amino acid sequence is from the RSV group A strain A2. The location of the CX3C motif in the G peptides is underlined. The amino acid sequence (393-405) for the L control peptide is IINGRWIILLSKFLK.

### Vaccination, Challenge and Tissue Collection

 BALB/c mice (4-6 wks old., The Jackson Laboratory) were housed in accordance with the guidelines of the CDC Institutional Animal Care and Use Committee. Mice were i.m. vaccinated with 10^6^ PFU equivalents of FI-A2 or FI-vero lysate. Groups of mice were then vaccinated with 50-75 µg of KLH-conjugated G peptides based on RSV A2 conserved regions, while a polymerase peptide was used as a negative control. Initial immunizations consisted of peptide emulsified 1:1 with TiterMax Gold (TiterMax) administered s.c while booster immunizations consisted of 50 µg peptide in sterile PBS administered i.p. at 14 and 28 days post-vaccination. Two weeks following final G peptide boost, the FI-RSV immunized mice, with or without the G peptide vaccination, were anesthetized by i.p. administration of Avertin (2% 2,2,2-tribromoethanol, 2% tert-amyl-alcohol, 180-250 mg/kg), and intranasally (i.n.) challenged with 10^6^ PFU of RSV virus, 10^6^ PFU RSV A2, equivalent PFUs of UV-inactivated RSV A2, or uninfected, vero-lysate control in SF-DMEM. UV-inactivated RSV A2 was confirmed inactivated by standard plaque assay.

After challenge, at the timepoints indicated, mice were anesthetized with Avertin and exsanguinated by severing the right brachial artery. Bronchoalveolar leukocyte (BAL) cells were harvested by lavaging the lungs with sterile Dulbecco’s PBS (D-PBS) and BAL cell numbers were counted. Lungs were removed and stored at -80°C until use. No fewer than three mice per treatment per time point were examined. 

### Histopathology

Formalin-fixed lung tissues and BAL cell morphology were determined by hematoxylin and eosin staining and light microscopy analysis as previously described [25]. BAL cells were fixed to cytoslides with 10% formaldehyde and stained with hematoxylin and eosin (H&E) as previously described. The percentages of lymphocytes, polymorphonuclear cells, macrophages and eosinophils were determined by examining morphological and staining features of 600 BAL cells per sample. 

### Flow Cytometry

The procedure used for extracellular staining of BAL cells was modified for microculture staining as described [26]. Briefly, BAL cells were blocked in 10% normal mouse serum in staining buffer (D-PBS with 1% bovine serum albumin) and then stained with the appropriate combinations of fluoroscein isothiocyanate-, allophycocyanin-, or phycoerythrin-labeled anti-CD3ε (T cell marker), anti-CD45R/B220 (B cell marker), anti-CD11b, anti-polymorphonuclear cell marker (RB6-8C5), anti-NK cell marker (DX5), and mouse isotype antibody controls (eBioscience). The distribution of cell surface markers was analyzed on a BD LSRII flow cytometer using FACSDiva software (Becton Dickinson, Mountain View, CA) from 10,000 lymphocyte-gated events. 

### Real-Time qRT-PCR

Total RNA was extracted from homogenized lung tissue using a RNA purification kit (QIAGEN, Valencia, CA) per manufacturer’s protocol and expression of the RSV M gene was determined by real-time qRT-PCR (Stratagene). Results are presented as relative genome level in PFU equivalents/ml and were measured against a RSV RNA standard curve.

### Cytokine analysis

Cell-free BAL supernatants were assayed for IFN-γ and IL-4 by multiplex bead based assay according to the manufacturer’s instructions (Bio-Rad) or by ELISA (eBioscience). Concentration of the cytokine was determined from a standard curve and data are presented as the mean pg/ml for individual mice for each group in duplicate. 

### ELISAs

Mouse sera were screened for whole RSV and anti-peptide antibody titers using a modified indirect ELISA as previously described [27]. Briefly, microtiter plates were coated with immunizing antigen overnight at 4°C. Serial dilutions of sera in PBS containing 5% milk and 0.1% Tween-20 were added to the well and incubated for 1 hr at 37°C. The plates were then washed with washing buffer (PBS containing 0.05% Tween-20) and incubated for 1 hr at 37°C with horseradish peroxidase-conjugated goat anti-mouse IgG (H+L, Millipore, Temecula, CA). After being washed, the plates were developed with ABTS (KPL) as indicated by the manufacturer, and the optical density (OD) was measured at 405 nm with a 490 reference filter. Whole RSV- specific antibody was detected using RSV-infected (positive antigen) or uninfected (negative antigen) Vero cell monolayers fixed onto the microtiter plates and developed using the procedure described above. 

### RSV microneutralization assay

 Heat-inactivated serum from immunized mice was used to determine neutralizing antibody titers as previously described [28]. Briefly, two-fold serial dilutions of pooled sera from 8 to 10 mice per pre-challenge vaccination group were mixed with equal volume RSV strain A2 (final concentration of 1X10^5^ PFU/well) and incubated for 1 hr at 37°C. After incubation, the virus-serum mixtures were added to microtiter plates containing 80% confluent monolayers of Vero cells, and incubated at 37°C for 2 hrs. After virus adsorption, the cell monolayers were overlaid with DMEM + 10% fetal bovine serum (DMEM+10%FBS), incubated at 37°C for three days, the monolayers fixed with a 80% to 20% acetone-PBS mixture, and immunostained with a pool of anti-F and anti-G protein mAbs (clones 131-2A, 131-2G, 130-6D) followed by horseradish peroxidase-conjugated goat anti-mouse IgG/IgM/IgA (H+L, KPL). Color was developed with ABTS (KPL) and OD measured at 410 nm. Each serum pool was assayed in triplicate, and the percent neutralization at each dilution was determined as the average value of ((OD_virus infection without antibody_ – OD_virus+serum antibody_)/OD_virus infection without antibody_)*100% for the three replicates. 

### Statistical Analysis

Data were analyzed for statistical significance using a Student’s *t* test, where a P value of <0.05 was considered statistically significant. Data are expressed as the mean + standard error of the mean (SEM).

## Results

### FI-RSV Enhanced Disease Model

**Figure 1:**
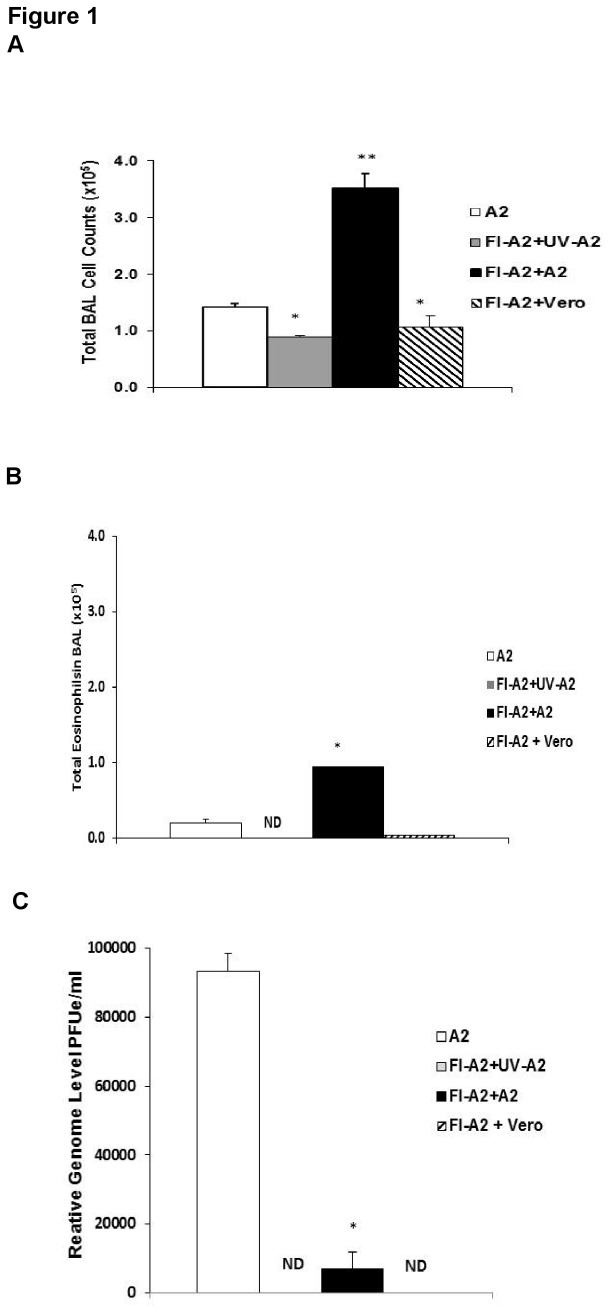
Vaccine enhanced disease in FI-A2 vaccinated mice after RSV Challenge. Mice were i.m. vaccinated with formalin inactivated RSV A2, rested for at four weeks and i.n challenged with either live RSV A2 (FI-A2+A2), UV-inactivated RSV A2 (FI-A2+UV-A2) or unifected vero cell lysate (FI-A2+vero). Unimmunized mice challenged with live RSV A2 (A2) provide a comparison to results after acute infection. Unvaccinated RSV challenged mice are compared to FI-RSV vaccinated mice challenged with live RSV mice and to FI-RSV vaccinated mice challenged with UV-inactivated RSV A2. Data from a separate experiment compares the inflammatory responses to FI-RSV vaccinated and vero cell lysate challenged mice. On day 5 post-infection, total BAL cell counts (A), total eosinophil counts (B) and viral RSV M gene mRNA (C) in the lungs of mice were determined. Total eosinophil counts were calculated by multiplying the total BAL cell counts by the percent eosinophils and presented as total eosinophils in BAL. Significance was calculated using a Student's *t* test, comparing mice in each group to mice vaccinated with FI-A2 alone and then challenged with RSV . * *p*<0.05 A2 compared to FI-A2+UV-A2 or FI-A2+vero; ** *p*<0.05 significance FI-A2 + A2 compared to either A2 or FI-A2+UV-A2 or FI-A2+vero. ND= not detected.

 In our FI-RSV model, peak inflammation occurs on day 5 post-challenge (p.c.) with live RSV [25] and we chose to present data on pulmonary inflammation for day 5. As illustrated in Figure 1, in this model of FI-RSV vaccination in mice and our method for preparing challenge virus the majority of the inflammation is not associated just with presence of viral antigens but requires live virus and, presumably, virus replication. In FI-RSV vaccinated mice, there was significantly fewer BAL cells (Figure 1A) and eosinophils (Figure 1B) after challenge with UV-inactivated RSV A2 or uninfected vero cell lysate than after live RSV challenge at day 5 p.i. Though infectious virus was not detected in RSV challenged, FI-RSV vaccinated mice (data not shown), low levels of RSV RNA were detected by real time PCR (Figure 1C). In this FI-RSV model, the marked increase in total number of cells and eosinophils in the BAL require challenge with live RSV challenge.

### Serum antibody response generated by vaccination

**Figure 2:**
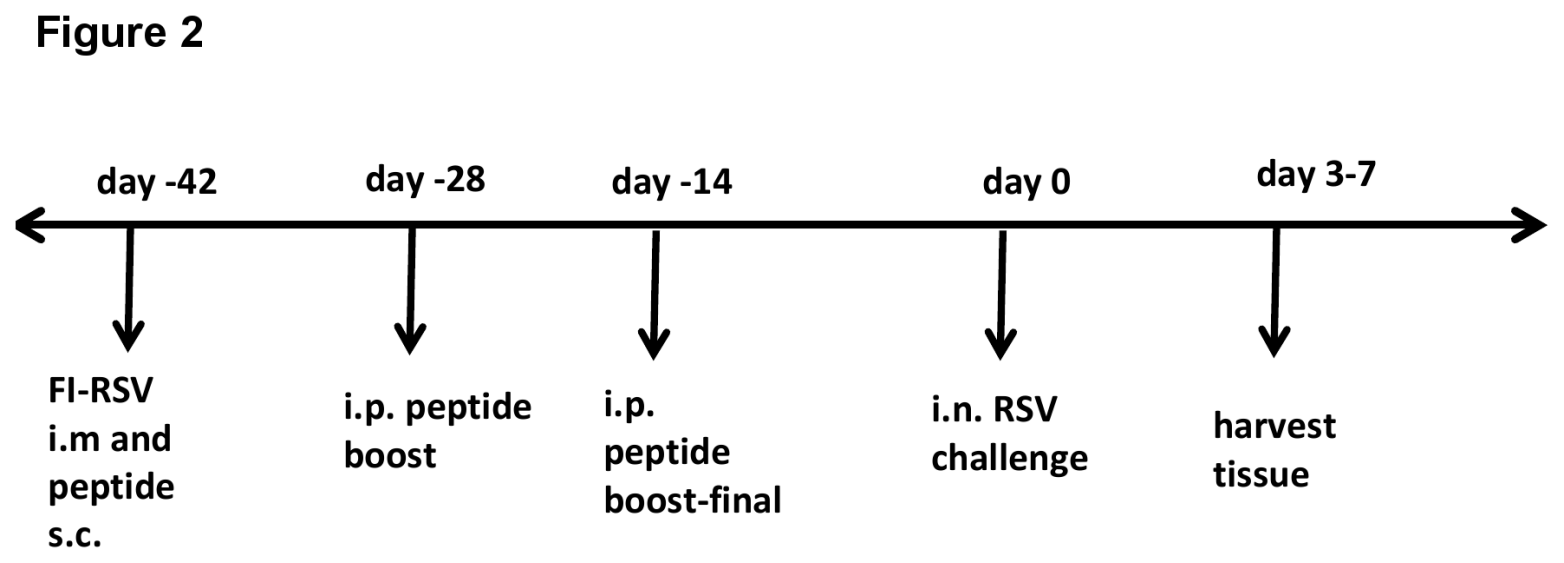
Immunization and challenge timeline. Timeline of vaccination and challenge schedule as described in the Materials and Methods.

**Figure 3:**
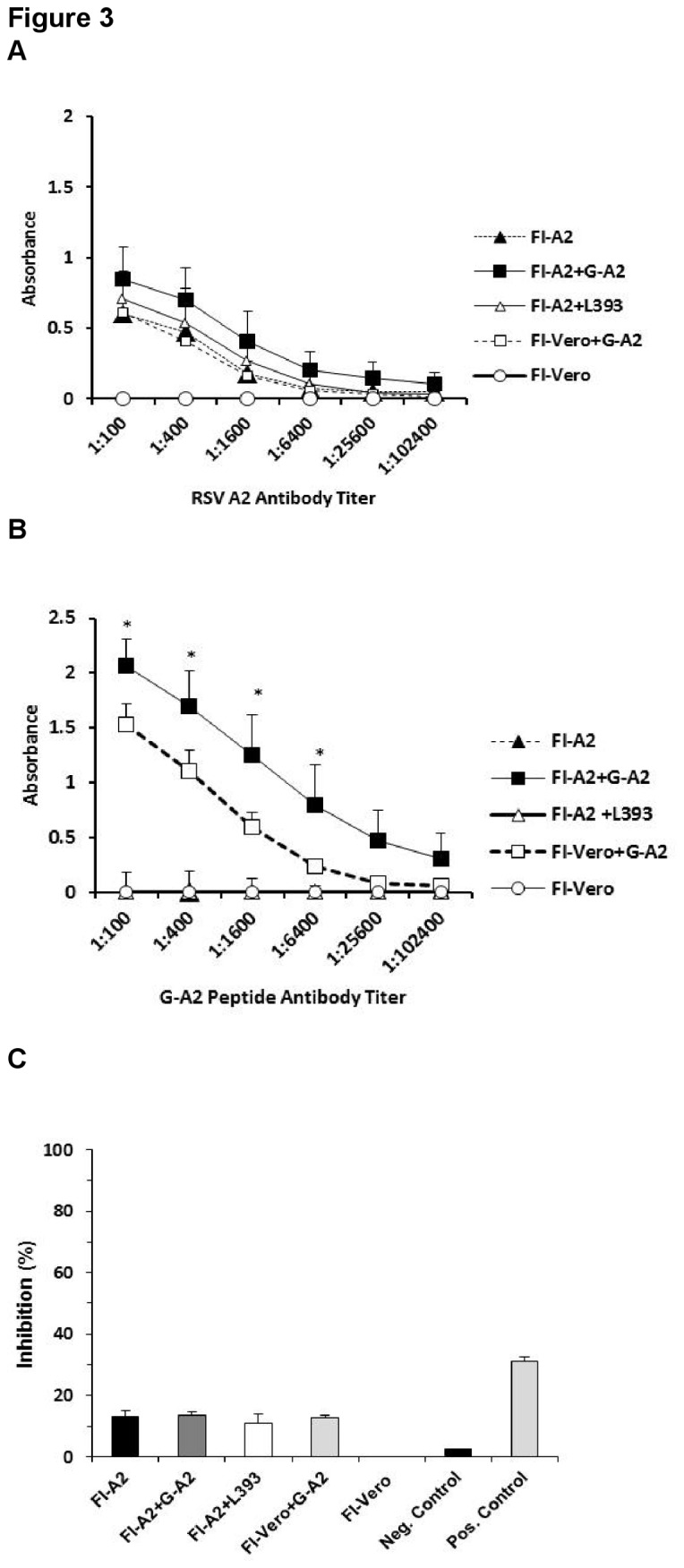
Antibody response against immunizing G peptides in FI-A2 vaccinated mice. Two weeks following the final boost and prior to RSV challenge, whole RSV A2 IgG specific titers (A) and G-A2 peptide specific IgG titers (B) in sera from mice vaccinated with formalin-inactivated RSV A2 alone (FI-A2), FI-A2 and RSV A2 G peptide (FI-A2+G-A2), FI-A2 and control L393 peptide (FI-A2+L393), FI-Vero alone (FI-Vero), or FI-vero and RSV A2 G peptide (FI-Vero+G-A2) were detected by indirect ELISA. Shown are the absorbance values (OD_405_) after subtraction of background OD_405_ values against control antigen. Results are means of 5 to 10 mice per group and are representative of three independent experiments. (C) Neutralizing antibodies against live RSV A2 in vaccinated animals (1:200 dilution) were detected by a microneutralization assay. Serum from a naïve mouse and anti-RSV F monoclonal antibody (143-6C) were used as negative and positive controls, respectively. * *p<0*.*05*.

The RSV G peptide, aa 163-190 of RSV A2 strain (G-A2), used in this study contained the G CX3C motif and is relatively conserved among the RSV Group A strains (Table 1). This G peptide was assessed for its ability to induce antibodies in the context of formalin-inactivated (FI)-RSV vaccination. BALB/c mice were i.m. vaccinated with either FI-A2 or FI-Vero control alone or subsequently immunized with G-A2 or control (L393) peptide antigens plus adjuvant. The mice receiving peptides were boosted twice with the respective peptides (Figure 2). Two weeks after the final boost, the anti-RSV A2 and G peptide-specific IgG titers were evaluated by an indirect ELISA assay (Figure 3). Mice immunized with FI-Vero control and a G-A2 peptide (FI-Vero+G-A2) vaccine developed antibodies against the whole virus (Figure 3A) and G-A2 peptide (Figure 3B); while mice immunized with FI-Vero alone (FI-Vero) had no detectable antibodies to either whole virus or G-A2 peptide. Vaccination with FI-RSV alone (FI-A2) or with G-A2 peptide (FI-A2+G-A2) induced IgG antibodies against whole virus (Figure 3A); however, only mice vaccinated with a RSV peptide developed G peptide specific-antibodies (Figure 3B). Similarly, FI-A2 vaccinated mice also vaccinated with the control peptide (FI-A2+L393) developed antibodies against the whole virus (Figure 3A) and control peptide (data not shown) but not the RSV G-A2 peptide. All FI-RSV and/or G-A2 peptide vaccinated mice developed low levels of neutralizing antibodies (Figure 3C). 

### Vaccination with RSV G peptides reduces FI-RSV vaccine enhanced disease

We have recently reported that vaccination with RSV G peptides containing the CX3C motif prevented body weight loss and pulmonary inflammation in BALB/c mice after acute RSV infection [29]. To evaluate whether RSV G peptide vaccination induced an immune response that also decreased FI-RSV vaccine enhanced disease, FI-RSV and G-A2 or L393 peptide vaccinated mice were challenged with RSV strain A2 two weeks after the final vaccination. Lung viral titers, pulmonary cell infiltration, body weight loss and histopathology were evaluated at various time points post- challenge. 

**Figure 4:**
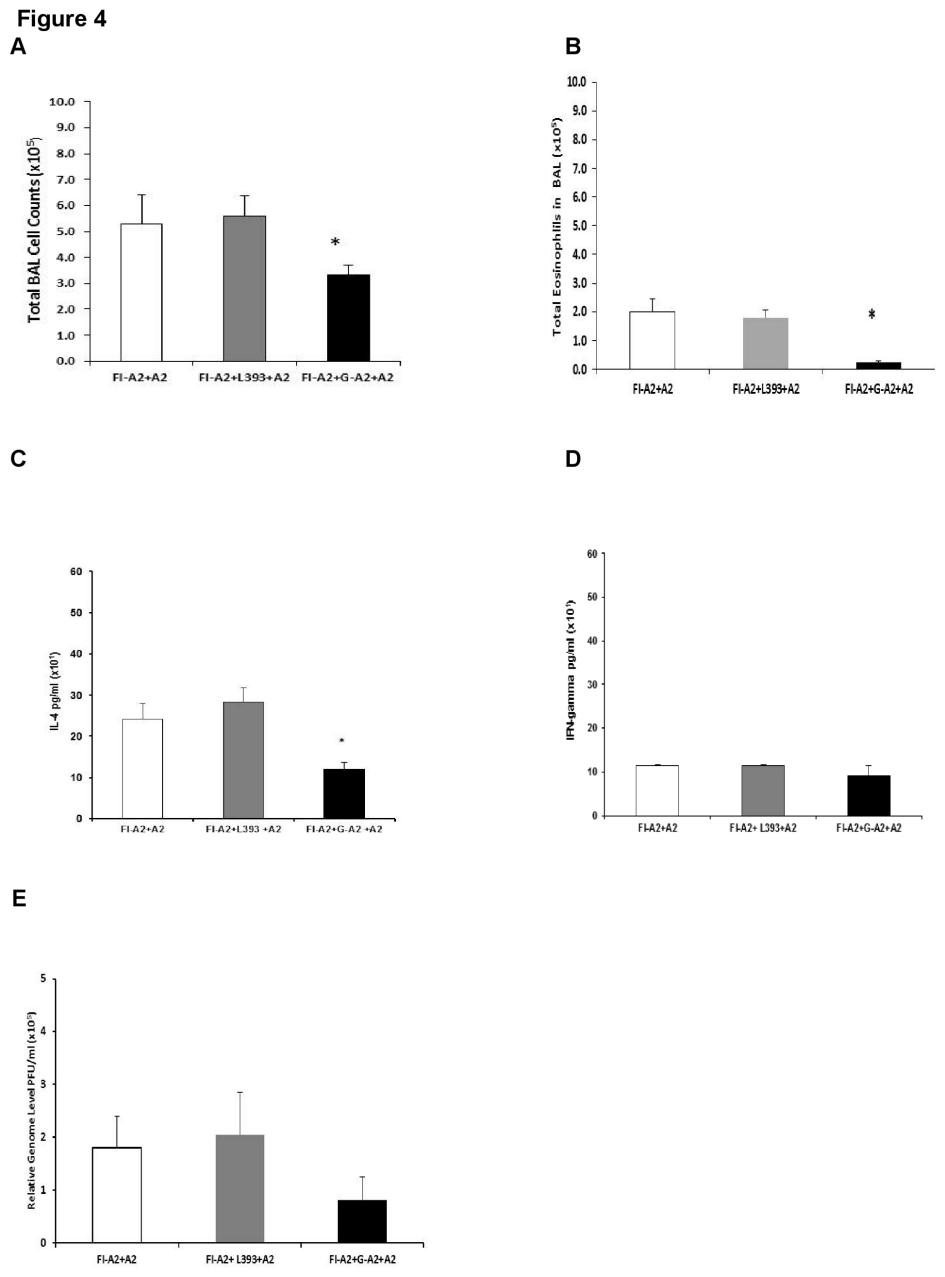
Decreased pulmonary cell inflammatory response in FI-A2 and G-A2 peptide vaccinated mice after RSV challenge. Mice were i.m. vaccinated with formalin inactivated RSV A2 (FI-A2) and then s.c. vaccinated with RSV A2 G peptide (FI-A2+G-A2) and control peptide (FI-A2 + L393). Two weeks following the final peptide boost, groups were challenged with live RSV A2 and the mean numbers of BAL cells/lung (A), total eosinophil counts in the BAL (B), IL-4 levels (pg/ml) (C) and IFN gamma (D) in the cell-free BAL supernatant, and viral message levels (relative genome equivalent PFU/ml) (E) at day 5 post-challenge (peak of cell infiltration) were determined. Representative data from five independent experiments are shown. * *p<0*.*05*.

**Figure 5:**
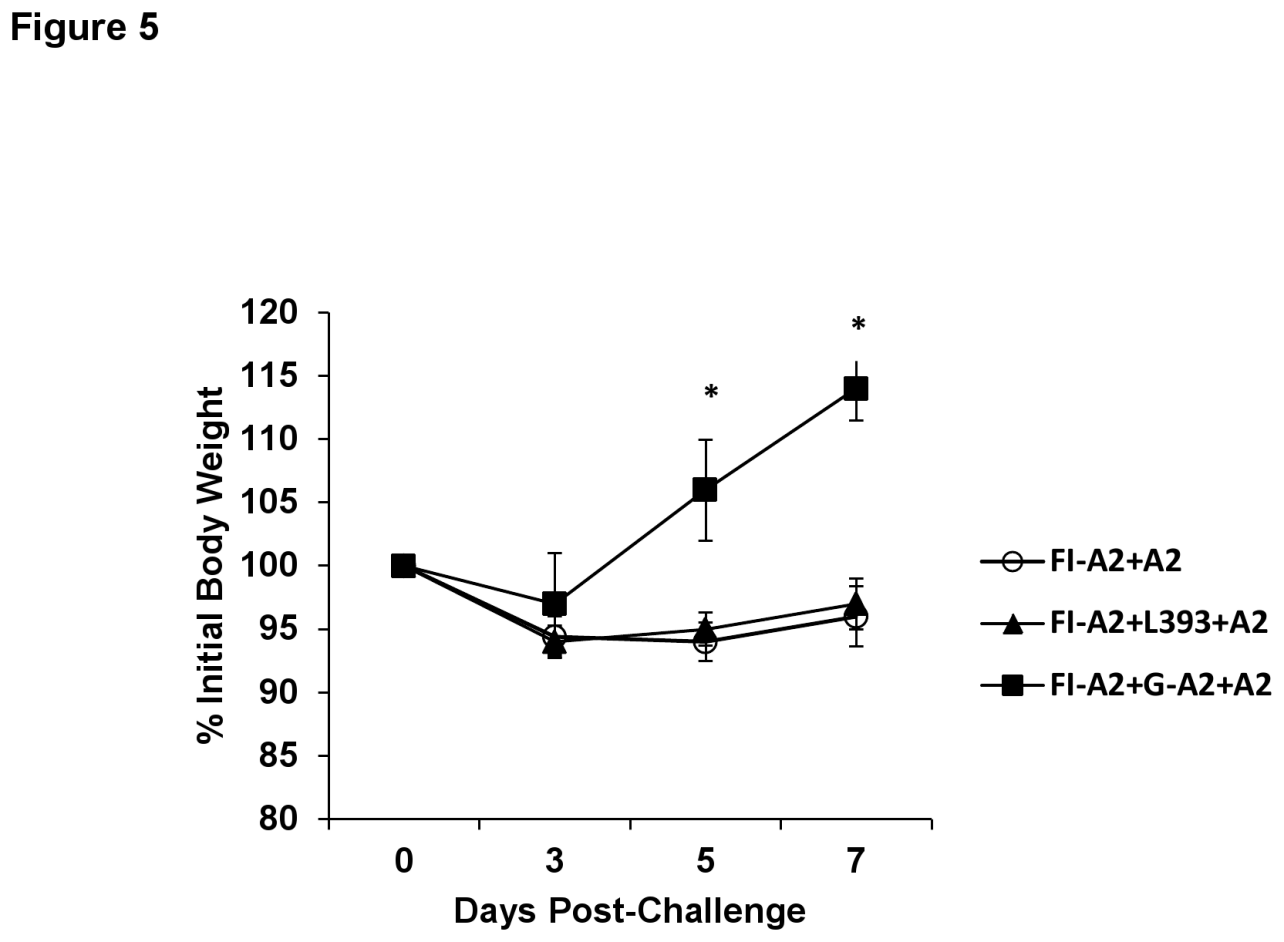
Decreased weight loss in FI-A2 and G-A2 peptide vaccinated mice after RSV challenge. Mice were i.m. vaccinated with formalin inactivated RSV A2 (FI-A2) and then s.c. vaccinated with G-A2 or control peptides. Two weeks following the final peptide boost, FI-A2 and RSV A2 G peptide (FI-A2+G-A2) and control L393 peptide (FI-A2+L393) mice were challenged with 10^6^ PFU live RSV A2 and percent body weight loss were determined. Five independent experiments were performed and combined (representative) data from two experiments are shown. * *p<0*.*05*.

As expected, mice that received FI-A2 alone developed substantial pulmonary BAL cell infiltration (Figure 4A) and eosinophilia (Figure 4B) following A2 challenge, as well as an increased Th2 type IL-4 cytokine response (Figure 4C), and increased body weight loss (Figure 5). Increased pulmonary Th2 type cytokine production and pronounced weight loss are associated with enhanced FI-RSV vaccine disease in mice [24,25]. In contrast, mice that received G-A2 peptide, but not control peptide, in addition to the FI-A2 vaccination, exhibited a significant decrease in this cellular inflammatory response (Figure 4A-C) that was seen as early as day 3 post-challenge (data not shown). Furthermore, the addition of the G-A2 peptide, but not control peptide, to FI-RSV vaccination was associated with a 32 to 56% decrease in all leukocyte subsets on day 5 post-challenge, with an even greater decrease (95%, p=0.002) in DX5^+^ cells (Table 2). Similar reductions in cell types were noted at day 3 and day 7 post-challenge in mice that received both FI-A2 and G-A2 peptide vaccination (data not shown). G-A2 peptide vaccination had minimal effect on Th1-type IFN-γ levels (Figure 4D). No infectious virus was detected by plaque assay of lung homogenates from any of the vaccinated mice but some viral replication, as measured by real time PCR, was detected. The viral message levels was similar for all vaccination schedules with a slight reduction (p=0.25) in the message levels after G-A2 peptide vaccination at day 5 post-challenge (Figure 4E). Weight loss was also significantly decreased (d5 p=0.011, d7 p=0.050) with the addition of the G-A2 peptide vaccine but not control peptide vaccine (Figure 5). 

**Table 2:** Pulmonary leukocytes in FI-RSV and G-A2 peptide vaccinated mice 5 days after RSV challenge^*a*^.

**Phenotype**	**FI-A2+ A2 (x10^3^)**	**FI-A2+G-A2 +A2 (x10^3^)**	
			**% Reduction**
**CD3**	146.1 + 20.2	**63.7 + 16.3**	**56**
**B220**	27.1+3.7	**14.9 + 3.8**	**45**
**DX5**	172.8+ 2.4	**8.6 + 2.2**	**95**
**CD11b**	55.9 + 7.7	**26.7 + 6.8**	**52**
**RB6-8C5**	31.1 + 4.3	21.1 + 5.4	32

^a^ Data represent the mean total BAL cells expressing CD3 (T cell marker), CD45R/B220 (B cell marker), DX5, (NK cell marker), CD11b (macrophage cell marker) or RB6-8C5 (PMN cell marker) from mice vaccinated with FI-A2 alone or with FI-A2 and G-A2 peptide, five days after RSV A2 challenge. The total number of BAL cells expressing a particular phenotype was determined by multiplying the mean total number of BAL cells by the mean percentage of cells expressing that phenotype. Boldface values indicate a significant decrease (*p<0.05*) in the number of BAL cells expressing a particular phenotype with addition of the G-A2 to FI-A2 vaccination.

Similar to G-A2 peptide vaccination, vaccination with RSV G peptides spanning the G CX3C motif of RSV strains NY/CH17/83 (aa 163-190; Group A specific) and B1 (aa 155-206; Group B specific conserved region) led to a significant decrease in the number of BAL cells and pulmonary eosinophils and a marked decrease in IL-4 levels (Figure S1) (p=0.015 for FI-B1 + G-B1 + B1; p=0.095 for FI-A2+G-CH17+ A2) and weight loss (data not shown) after RSV challenge in FI-RSV vaccinated mice. 

**Figure 6:**
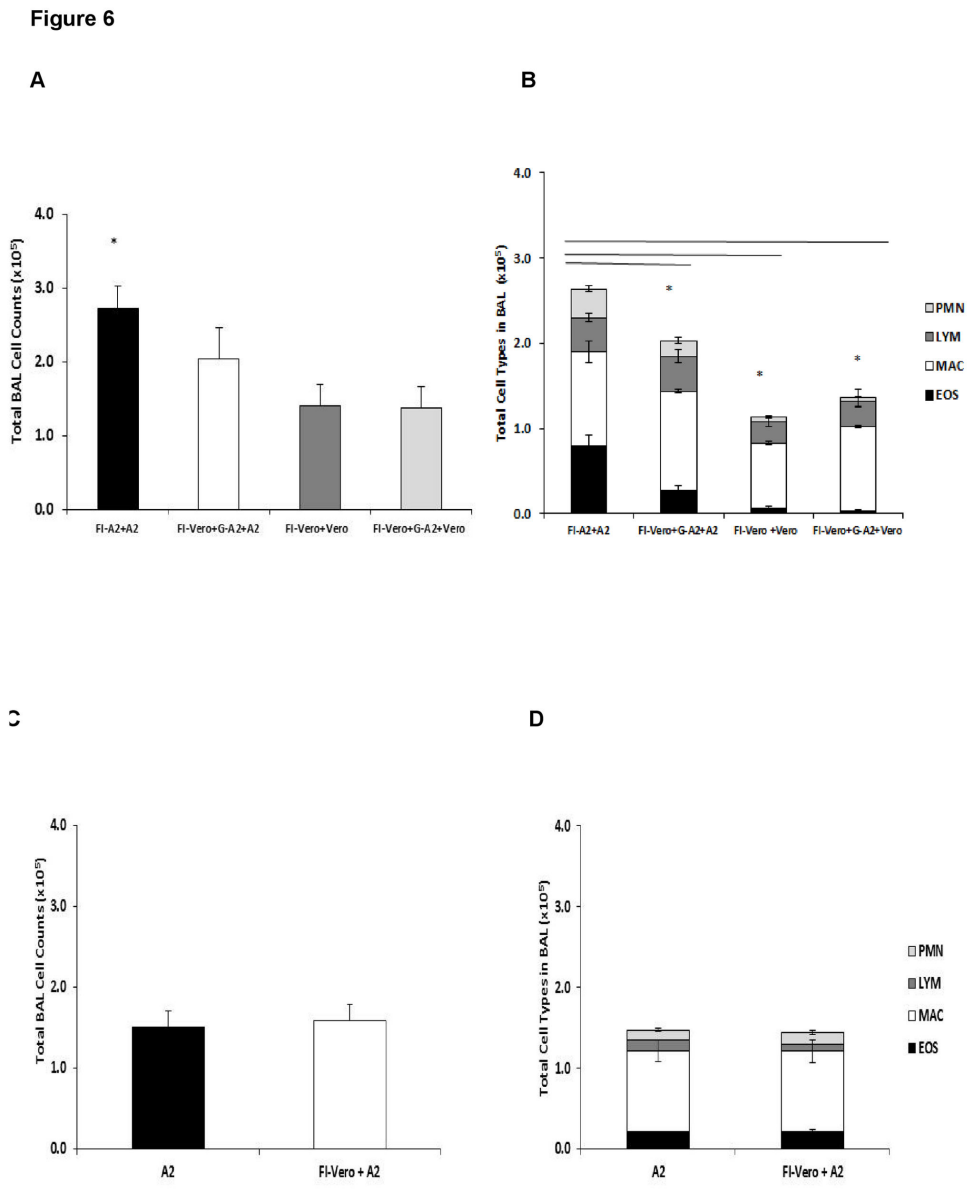
Pulmonary cell infiltration following FI-RSV and FI-Vero vaccination. (A) Total BAL cell counts in the lungs of mice i.m. vaccinated with FI-RSV A2 and live RSV A2 challenged (FI-A2+A2), mice vaccinated with FI-Vero cell lysate supernatant and then challenged with Vero cell lysate supernatant (FI-Vero+Vero), or mice vaccinated with FI-Vero lysate and G-A2 peptide and challenged with either RSV A2 (FI-Vero+G-A2+A2) or uninfected, Vero lysate (FI-Vero+G-A2+Vero) (day 5 post-infection). (B) Total neutrophils (PMN), lymphocytes, (LYM), macrophages (MAC) and eosinophils (EOS) counts in BAL. (C) Total BAL cell counts in the lungs of mice vaccinated with or without FI-Vero supernatant at day 5 post-RSV A2 challenge. (D) Total PMN, LYM, MAC and EOS counts in the BAL of mice infected with A2 and mice vaccinated with FI-Vero cell lysate and then challenged with RSV A2 (FI-Vero+A2) at day 5 post-challenge are shown. Significance was calculated using a Student's *t* test, comparing mice in each group to mice vaccinated with FI-A2 alone and challenged with RSV. * *p<0*.*05*.

The cellular response to various controls showed that most of the findings noted above are associated with a response to live RSV and to a lesser extent cellular or other components in the challenge preparation (Figure 6). At the peak of cell infiltration, day 5 post-challenge, the total BAL numbers (Figure 6A) and type of BAL cells (Figure 6B) in the lungs from various Vero cell control vaccinations and challenges (FI-Vero+Vero, FI-Vero+G-A2+Vero) were substantially less than in FI-RSV vaccinated, RSV A2 challenged mice (FI-A2+A2) and similar to that for FI-A2 vaccinated mice given G-A2 peptide (FI-A2+G-A2+A2) (Figure 6 A and 6B). These differences between FI-A2 vaccinated, RSV A2 challenged mice and various controls suggests that elimination of fetal calf serum from the challenge virus, as expected, decreased the non-RSV component of the inflammatory response in these studies. Mice immunized with FI-Vero and the G-A2 peptide, then challenged with RSV A2 (FI-Vero+G-A2+A2) had slightly higher BAL cell numbers (p=0.161) (Figure 6A) and eosinophil infiltration (p=0.084) (Figure 6B) than the other control groups, but significantly less than what was observed in FI-RSV vaccinated, RSV A2 challenged mice. This slight increase in pulmonary inflammation after the G-A2 peptide alone may have resulted in part from a lower peptide-specific antibody response without the FI-RSV vaccination (Figure 3B). Note in Figure 3B that the anti-G-A2 peptide antibody levels were significantly lower after FI-Vero plus G-A2 peptide vaccination (FI-Vero+G-A2) than after FI-RSV plus G-A2 peptide vaccination (FI-A2+G-A2). FI-Vero immunization and RSV A2 challenge (FI-Vero+A2) induced BAL cell infiltration similar to levels seen in unimmunized, RSV A2 infected mice (Figure 6C-D). 

**Figure 7:**
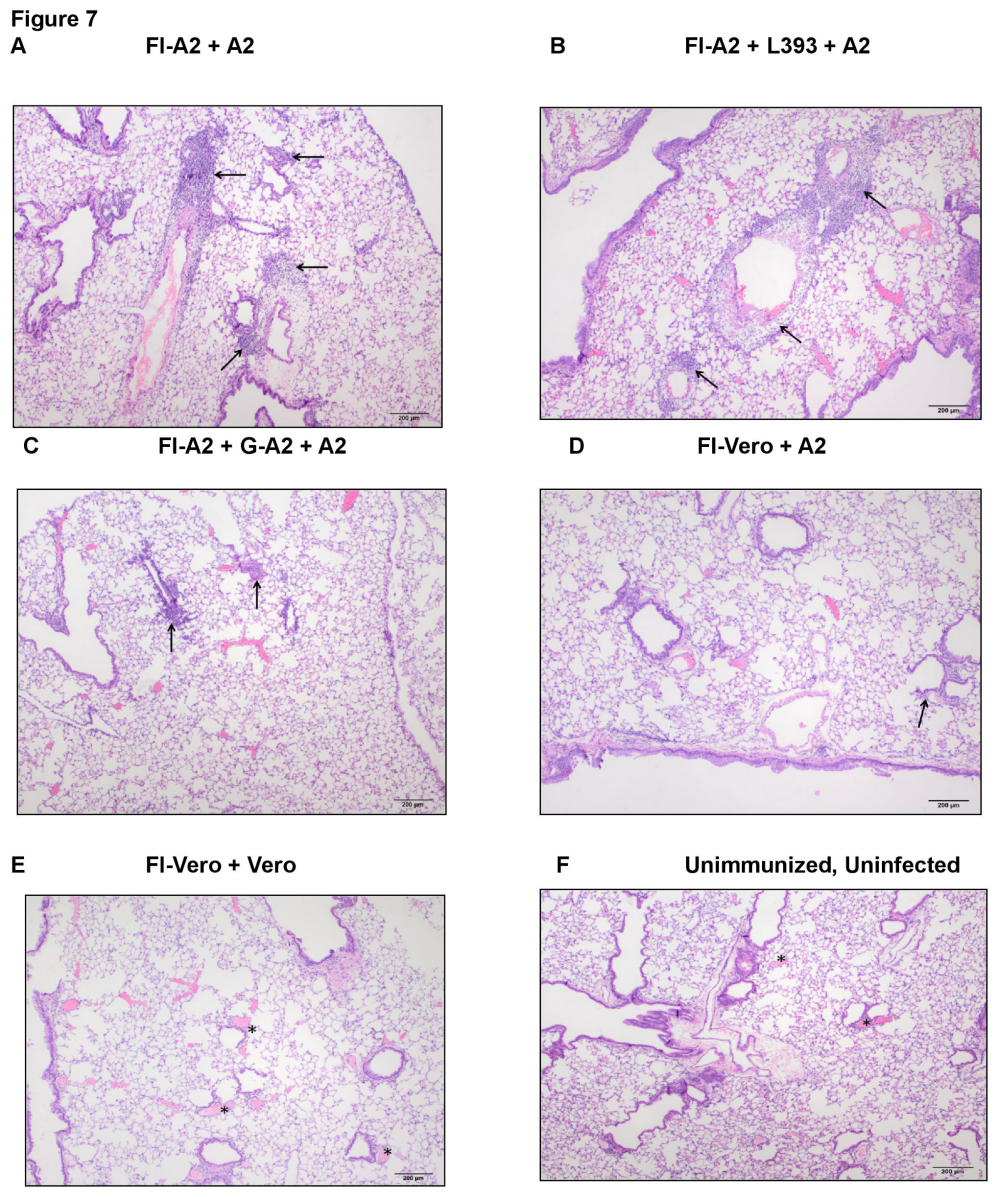
Lung histopathology in vaccinated mice. Lung histopathology was examined by hematoxylin and eosin staining in (A) mice i.m. vaccinated with FI-A2 and then challenged with RSV A2 (FI-A2), (B) mice vaccinated with FI-A2 and control L393 peptide and challenged with RSV A2 (FI-A2+L393), or (C) mice vaccinated with FI-A2 and G-A2 peptide and challenged with RSV A2 (FI-A2+G-A2) at day 3 post-challenge. The arrows mark areas in inflammatory infiltrates. Control groups were immunized with FI-Vero cell lysate and challenged with either (D) RSV A2 (FI-Vero) or (E) uninfected, Vero lysate (FI-Vero+Vero). (F) Lung histopathology of an uninfected, unimmunized BALB/c mouse. The perivascular areas (asterisks) are devoid of inflammatory infiltrates. 40X original magnification, Scale bar= 200mm.

The degree of lung histopathology associated with FI-RSV enhanced disease was evaluated. By day 3 post- challenge, mice vaccinated with FI-A2 alone (Figure 7A) or with the control peptide (Figure 7B) had heavy perivascular and peribronchiolar cellular infiltration consisting of large numbers of lymphocytes, and eosinophils, typical of FI-A2 vaccine enhanced disease, whereas the addition of G-A2 peptide vaccination resulted in substantially less (mild) lung infiltration (Figure 7C). The lung histopathology in FI-Vero vaccinated and RSV challenged mice was mild to moderate (Figure 7D), while the lungs of FI-Vero vaccinated and mock-infected mice had normal histology comparable to that of unimmunized, uninfected mice (Figure 7E-F). Taken together, these data suggest that vaccination with a RSV G peptide encompassing the Group A or Group B conserved region has the ability to decrease enhanced pulmonary inflammation associated FI-RSV vaccination.

## Discussion

RSV continues to be a high priority for vaccine development. Unfortunately, the decades of work on developing vaccines have not identified a clear path to success. The severity of enhanced disease with natural infection in children given FI-RSV vaccines in the 1960s has blocked one specific approach to RSV vaccine development. It raises, and makes it difficult to allay, concerns about the safety of any non-live virus vaccine for RSV naïve children. The findings in the present study suggest that a new approach to non-live RSV virus vaccines is worth considering. Our results show that appropriately designed RSV G peptide vaccines can prevent much of the FI-RSV vaccine enhanced disease in mice. A RSV G peptide vaccine that substantially decreases FI-RSV enhanced disease in this animal model is unlikely to present a risk of enhanced disease in humans and could possibly be used with other candidate subunit vaccines to make them safer. Another RSV G peptide vaccine, BBG2Na, has been studied in animals and adults [30,31]. The peptide, which encompassed aa 130-230 of RSV G was larger than the ones we studied, and the vaccine included the albumin-binding domain of streptococcal G as an adjuvant. The vaccine was effective in animals, showed no enhanced disease with later RSV infection in humans, but adverse reactions at the time of vaccination led to discontinuation of clinical studies. These adverse events were presumably caused by adjuvant and not the RSV G peptide.

In the present study, the peptides were selected based on the amino acid sequences of the RSV G; they are relatively conserved among RSV isolates and include the binding site for mAb 131-2G and the CX3C motif. There are two relatively conserved amino acid sequences within the RSV G, one for group A and one for group B strains that include the CX3C motif and are large enough to be immunogenic. A vaccine that includes both of these group A (28 a.a.) and group B (52 a.a.) conserved region peptides should be capable of inducing antibodies against all known RSV strains and could protect from both natural and vaccine associated enhanced disease. A similarly constructed RSV G peptide vaccine that spans the central conserved region was recently shown to decrease pulmonary inflammation seen after primary RSV infection [29]. 

One major concern about any RSV G based vaccine is based on studies that have shown that vaccination with intact or secreted RSV G can cause enhanced inflammation, including eosinophilia upon RSV challenge [16-18,32]. In this study, RSV G peptide vaccine, when given at the same time as FI-RSV vaccination led to significant decreases in four measures of enhanced disease; i.e. pulmonary cell infiltration, eosinophilia, IL-4 levels and weight loss. The lack of enhanced inflammation after peptide vaccination may be due to the vaccine’s ability to effectively induce antibodies that block RSV G binding to CX3CR1 and/or because they do not contain regions of RSV G that induce disease enhancing responses. To alleviate any concerns about an RSV G peptide vaccine, however, it will be necessary to understand the mechanisms by which this vaccine reduced FI-RSV vaccine enhanced disease in mice, determine if these findings apply to humans and clarify differences in immunity induced by peptide vaccines, intact G, and secreted G. We hypothesize that antibodies induced by the peptide vaccines bind to RSV G and prevent RSV G associated disease during infection which in turn decreased FI-RSV enhanced disease. This idea follows from our previous studies showing that altering the ability of RSV G to bind to CX3CR1 by using a virus lacking RSV G [25], a virus with a mutation in the CX3C site [25], or by blocking RSV G binding to CX3CR1 using anti-G or anti-CX3CR1 mAbs markedly decreased measures of enhanced disease [24,25,26,33]. These studies suggest that the RSV G, in particular the CX3C motif, contributes to the enhanced inflammatory response to FI-RSV vaccination upon RSV challenge. These studies show a role for RSV G at the time of virus challenge but not a role for RSV G at the time of FI-RSV vaccination. The hypothesis that enhanced disease prevented by RSV G peptide immunization results from induction of antibodies that bind to RSVG during RSV infection is supported by data showing anti-G peptide antibodies only in mice vaccinated with both FI-RSV and RSV G peptide. Another possible explanation for the reduced inflammation seen after peptide vaccination is a reduction in virus replication. The RSV message levels detected in the lungs of FI-RSV vaccinated mice were low and slightly lower for FI-RSV mice also given the RSV G peptide vaccine but not significantly so. 

The possibility that peptide vaccination might be used to modify the cellular immune response to RSV challenged after FI-RSV vaccination has not previously been considered. Induction of a Th2-type biased adaptive immune response by FI-RSV is considered a likely contributor to the enhanced disease with later infection [25,34-38]. Generation of reactive carbonyl groups during formalin inactivation of the virus may be responsible for inducing this Th2-biased response [39]. Alternatively, FI-RSV enhanced disease has been associated with induction of an overabundance of non-neutralizing antibodies compared to neutralizing antibodies and deposition of immune complexes [40]. Most attempts to address enhanced disease have focused on the vaccine and eliminating induction of the Th2-bias associated with FI-RSV enhanced disease and sometimes seen with other subunit RSV vaccines [41-43]. The present study suggests that the administration of RSV G peptides that induce antibodies to the central conserved region of RSV G may decrease enhanced disease, not by altering immunity induced by the vaccine but by binding RSV G and altering its role at the time of RSV challenge. We suspect that a sufficiently immunogenic RSV G peptide vaccine might decrease inflammatory responses associated with natural infection following any RSV vaccine.

In conclusion, our data demonstrate that an appropriately designed RSV G peptide vaccine can prevent enhanced disease under the most challenging circumstances, i.e. FI-RSV vaccination, and consequently suggest use of a RSV G peptide vaccine to improve the safety profile of other RSV vaccines merits further study. It is possible that co-vaccination with a RSV G peptide could provide the safe use of a non-live virus vaccine in the RSV naïve child. These data also support a role for the RSV G in the challenge virus in FI-RSV enhanced disease and are another approach to clarifying the pathogenesis of enhanced disease. RSV G peptide vaccines and their potential contribution to an RSV vaccine merits further study. 

## Supporting Information

Figure S1 **Decreased pulmonary cell inflammatory response in FI-A2 or FI-B1 and RSV G-CH17 or G-B1 peptide vaccinated mice after RSV challenge.** Mice were i.m. vaccinated with 10^6^ PFU equivalents of formalin inactivated (FI) RSV A2 (FI-A2) or FI-RSV B1 and then s.c. vaccinated with RSV CH17 G peptide (FI-A2+G-CH17) or RSV B1 G peptide (FI-B1+G-B1) as described in the Materials and Methods. Two weeks following the final boost with respective peptides, groups were challenged with either live RSV A2 (FI-A2+G-CH17) or RSV B1 (FI-B1+G-B1) and mean numbers of BAL cells/lung (A-B), total eosinophil counts in the BAL (C-D), and IL-4 levels (pg/ml) in the cell-free BAL supernatant (E-F) were determined. Error bars represent the SEM. Significance was calculated using a Student's *t* test comparing mice vaccinated with FI-RSV alone and mice vaccinated with both FI-RSV and respective RSV G peptide at indicated time points after RSV challenge. Representative data from three independent experiments are shown. * *p<0.05* G-CH17 peptide represents the amino acid sequence (aa 163-190) for the RSV group A strain CH17 (FHFEVFNFVPCSICSNNPTCWDICKRIP), and G-B1 peptide (PPKKPKDDYHFEVFNFVPCSICGNNQLCKSICKTIPSNKPKKKPTIKPTNKP) represents the amino acid sequence (aa 155-206) for the RSV group B strain B18537.
(TIF)
