# Supplementary figures and images for: Decrease in Formalin-Inactivated Respiratory Syncytial Virus (FI-RSV) Enhanced Disease with RSV G Glycoprotein Peptide Immunization in BALB/c Mice
Source: PLoS One. 2013 Dec 23;8(12):e83075. doi: 10.1371/journal.pone.0083075 (PMC3871585; doi:10.1371/journal.pone.0083075)

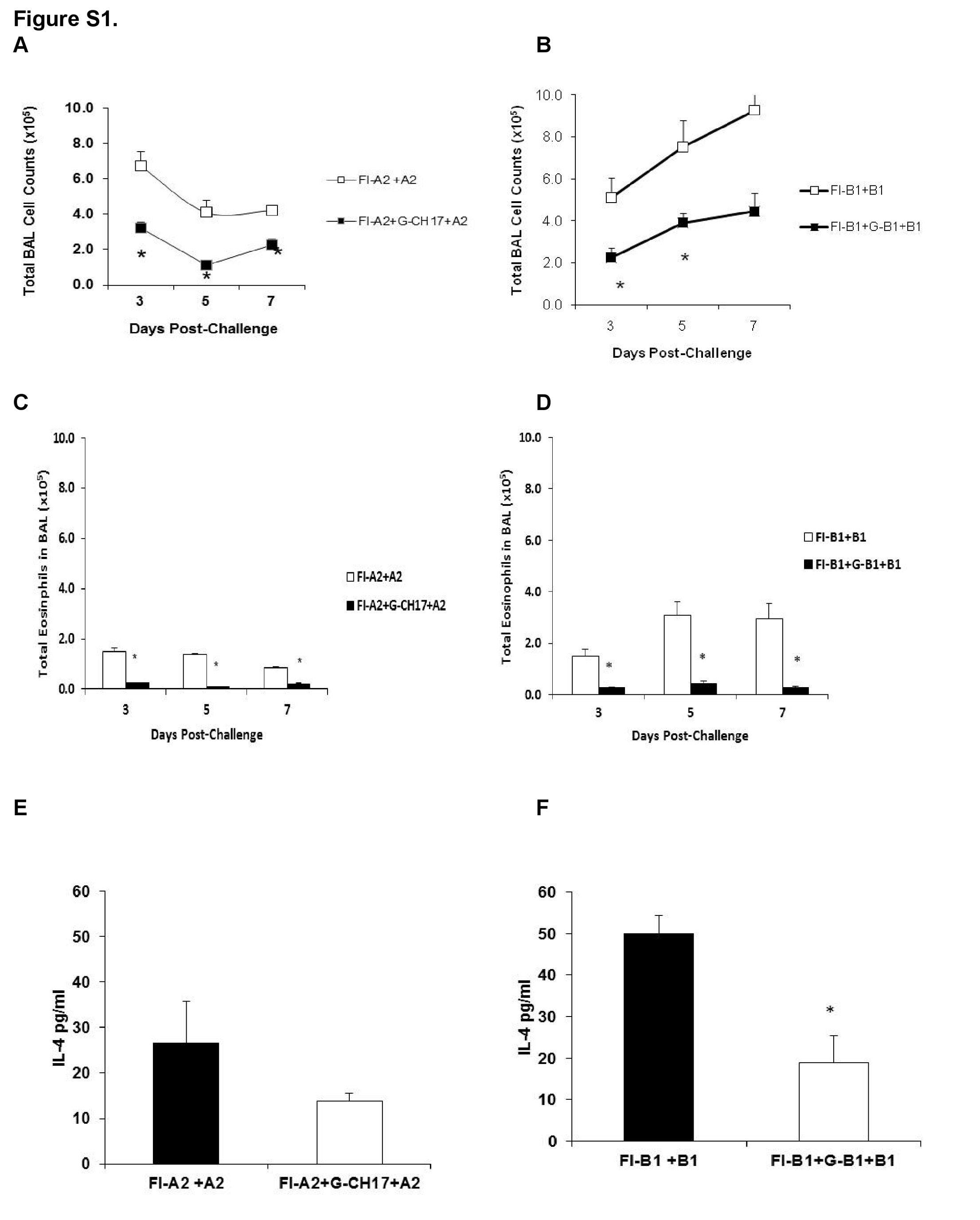

Supplement: Figure S1 — Decreased pulmonary cell inflammatory response in FI-A2 or FI-B1 and RSV G-CH17 or G-B1 peptide vaccinated mice after RSV challenge. Mice were i.m. vaccinated with 106 PFU equivalents of formalin inactivated (FI) RSV A2 (FI-A2) or FI-RSV B1 and then s.c. vaccinated with RSV CH17 G peptide (FI-A2+G-CH17) or RSV B1 G peptide (FI-B1+G-B1) as described in the Materials and Methods. Two weeks following the final boost with respective peptides, groups were challenged with either live RSV A2 (FI-A2+G-CH17) or RSV B1 (FI-B1+G-B1) and mean numbers of BAL cells/lung (A-B), total eosinophil counts in the BAL (C-D), and IL-4 levels (pg/ml) in the cell-free BAL supernatant (E-F) were determined. Error bars represent the SEM. Significance was calculated using a Student's t test comparing mice vaccinated with FI-RSV alone and mice vaccinated with both FI-RSV and respective RSV G peptide at indicated time points after RSV challenge. Representative data from three independent experiments are shown. * p<0.05 G-CH17 peptide represents the amino acid sequence (aa 163-190) for the RSV group A strain CH17 (FHFEVFNFVPCSICSNNPTCWDICKRIP), and G-B1 peptide (PPKKPKDDYHFEVFNFVPCSICGNNQLCKSICKTIPSNKPKKKPTIKPTNKP) represents the amino acid sequence (aa 155-206) for the RSV group B strain B18537. (TIF) [file pone.0083075.s001.tif]
